# A theoretical approach to zonation in a bioartificial liver

**DOI:** 10.1002/bit.23279

**Published:** 2012-01

**Authors:** Adam J Davidson, Marianne J Ellis, Julian B Chaudhuri

**Affiliations:** Centre for Regenerative Medicine, Department of Chemical Engineering, University of BathBath BA2 7AY, UK

**Keywords:** bioartifical liver, zonation, modeling, hepatocyte

## Abstract

Bioartificial livers have yet to gain clinical acceptance. In a previous study, a theoretical model was utilized to create operating region charts that graphically illustrated viable bioartificial liver configurations. On this basis a rationale for the choice of operating and design parameters for the device was created. The concept is extended here to include aspects of liver zonation for further design optimization. In vivo, liver cells display heterogeneity with respect to metabolic activity according to their position in the liver lobule. It is thought that oxygen tension is a primary modulator of this heterogeneity and on this assumption a theoretical model to describe the metabolic zonation within an in vitro bioartificial liver device has been adopted. The distribution of the metabolic zones under varying design and operating parameters is examined. In addition, plasma flow rates are calculated that give rise to an equal distribution of the metabolic zones. The results show that when a clinically relevant number of cells are contained in the BAL (10 billion), it is possible to constrain each of the three metabolic zones to approximately one-third of the cell volume. This is the case for a number of different bioreactor designs. These considerations allow bioartificial liver design to be optimized.

## Introduction

Liver disease is a prevalent and serious illness throughout the developed world. According to data from the Office of National Statistics in the UK, it is the fifth biggest killer and the only major cause of death to increase year-on-year. Acute liver failure in particular is a challenging condition to manage where survival without a liver transplant is as low as 20% (Craig et al., 2010). The range of different causes for liver failure and resulting variations in disease progression limit treatment options with liver transplant normally being the only viable choice (Lee et al., 2008). Organ transplantation is effective, but the shortage of donor organs and high demand has motivated research towards an alternative treatment.

In bioartificial livers (BALs) blood or plasma from the patient interacts with cells contained in an extracorporeal bioreactor. The cells are either primary liver cells or liver-like cell lines that aim to support or replace the patient's liver function. Many different designs of BAL have been suggested, though relatively few have progressed beyond Phase I clinical trials in humans (Wigg and Padbury, 2005). Those that do reach this stage have so far failed to demonstrate they significantly increase patient survival rates (Demetriou et al., 2003; Sauer et al., 2003; van de Kerkhove et al., 2004).

**Figure 1:**
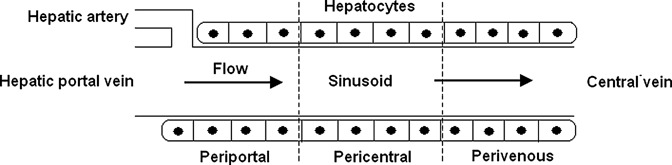
Schematic of the liver microcirculation. The gradient of oxygen tension along the sinusoid is a primary modulator of liver function and delineates the three metabolic zones.

BAL device design needs to be improved in order to progress to positive results and clinical acceptance. One aspect that has not been considered fully in BAL designs to date is the phenomenon of liver zonation. As blood travels through the liver microcirculatory system, or sinusoids, the concentration of substrates such as oxygen or hormones will change, causing the signals received by the hepatocytes in different regions of the lobule to also differ (Fig. 1). As a result hepatocyte metabolism and gene expression becomes dependent on the cell's position along the liver lobule. This heterogeneity in cell function according to position is the definition of liver zonation. The cells in the periportal zone, the region close to branches of the portal vein and hepatic artery, differ from those in the perivenous zone, which is the region close to the central vein. These differences include the presence of enzymes and receptors in the cells (Kietzmann and Jungermann, 1996). An article by Jungermann and Kietzmann (2000) explains the functional differences between the periportal and perivenous zones. Examples of processes that are prevalent in the periportal zone are bile formation and urea synthesis whereas glutamine formation and xenobiotic metabolism take place in the perivenous zone.

Liver zonation plays a major role in the overall function of the liver. A good example of this is the liver's control of blood sugar levels, that is, the glucostat function. During the postabsorptive phase (between meals) when glucose is scarce, periportal hepatocytes convert glycogen to glucose which in turn becomes lactate in the perivenous cells. The lactate enters the circulation and returns to the periportal zone, where enzymes involved in gluconeogenesis are upregulated to convert it to glycogen. During the absorptive phase, glucose is unutilized by the periportal zone and travels directly to the perivenous hepatocytes where insulin converts it to glycogen for storage (Allen and Bhatia, 2003; Jungermann and Kietzmann, 2000; Yu et al., 2009).

Zonation in the liver is generally thought to be primarily modulated by gradients of oxygen, hormones, and extracellular matrix components in the liver lobule. In particular, oxygen is found to be a modulator of both short-term metabolism and long-term gene expression in vivo (Gebhardt, 1992; Jungermann and Kietzmann, 2000; Kietzmann and Jungermann, 1996). Within a bioreactor, oxygen gradients will be determined by cellular uptake, operating conditions and bioreactor geometry. It is hypothesized that if these aspects can be controlled so as to produce a physiological oxygen gradient within the bioreactor, the hepatocytes will exhibit similar behavior as in vivo. In one study, it was found that a physiological-like oxygen gradient in a flat-plate bioreactor containing rat hepatocytes contributed to heterogenous distribution of phosphoeonolpyruvate carboxykinase (PEPCK) and cytochrome P450 2B (CYP 2B). The spatial patterning of these proteins in the bioreactor correlated well with their in vivo zonation (Allen and Bhatia, 2003). In a subsequent article, rat hepatocytes were co-cultured with non-parenchymal cells in a flat-plate bioreactor under a physiological oxygen gradient. The spatial induction of CYP 2B and CYP 3A was examined and found to mimic in vivo zonation (Allen et al., 2005). These results confirm that oxygen is a primary modulator of liver zonation both in vivo and in vitro.

Here we describe a model to simulate the effect of oxygen gradients in controlling liver zonation. A previous study found that by defining two operating constraints on a BAL design, operating and design parameters can be controlled in order to produce a viable device (Davidson et al., 2010). This work extends that concept to include liver zonation and how the size of each metabolic zone can be influenced within the range of viable operating parameters and fiber geometry. It was found that while the relative size of each metabolic zone varied over a large range, the distribution of the three metabolic zones could be controlled. In certain cases each zone can be made to occupy the same volume of the BAL, that is, a third. In addition, consequences arising from hyper-oxygenating the cell mass are revealed.

## Theoretical Aspects

A common feature of many BALs, for example the HepatAssist (Rozga et al., 1992) or ELAD (Sussman et al., 1991), is the use of hollow fibers. For this reason, this work considers a hollow fiber bioreactor (HFBR) model for use as a BAL. To model a HFBR, a common approach is to utilize Krogh cylinders (Abdullah et al., 2009; Chen and Palmer, 2010; Krogh, 1919; Patzer, 2004). Geometric symmetry is assumed to reduce the problem from a fiber bundle to a single fiber surrounded by an annulus of extracapillary space. The three regions of the Krogh cylinder represent the fiber lumen, fiber membrane, and the cell layer on the outer surface of the fiber.

The high surface area to volume ratio of hollow fibers allows larger cell populations to be cultured, and the fibers themselves can act as barriers to fluid shear stress or an immune response. However, as plasma/liver cell interaction occurs across a membrane, mass transport limitations can restrict the viability of such a device. Oxygen is generally regarded as being the most critical nutrient in hollow fiber bioreactor applications (Piret and Cooney, 1991). This is a particularly difficult issue in BALs as liver cells are highly metabolic and consume oxygen at around 10 times the rate of most other cell types (Cho et al., 2007). Oxygen transport in the BAL is modeled through the convection/diffusion equation (Equation 1). This equation has to be solved in each of the three regions of the Krogh cylinder. The forms of these equations and the associated boundary conditions are described more thoroughly in a previous study (Davidson et al., 2010).

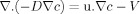
1
all symbols are defined in the Nomenclature section.

The convection-diffusion equations are solved using Comsol 3.5a (Comsol AB), a multiphysics software package. The Krogh cylinder is drawn in 2-d polar coordinates and discretized by finite elements, numbering over 4000. As the Krogh cylinder is of a very high aspect ratio, meshing of the structure is problematic unless the axial coordinates are scaled. In this case the axial lengths are scaled by a factor of 1000, which is representative of typical HFRB aspect ratios. This scaling requires adjustments to be made to the governing equations of the system—the diffusion coefficient becomes anisotropic, 10^6^ times smaller in the axial direction, and the axial flow velocity is scaled down by a factor of 10^3^. This procedure does not affect the results produced.

In our previous work, an operating constraint was defined for the BAL: oxygen levels should not fall below 2 mm Hg as this would lead to cell necrosis (De Groot et al., 1988). This constraint results in a definition for a maximum allowable number of hollow fibers in the BAL (*N*_max_) for a given device plasma flow rate *Q* (Equation 2), where *M*_2_ and *C*_2_ are empirical constants obtained from the modeling results, relating position of the hypoxic zone to fiber lumen radius. The subscript 2 denotes the partial pressure of oxygen being referred to, in this case 2 mm Hg to represent hypoxia.

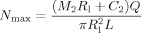
2

A minimum number of hepatocytes are required to achieve the desired device functionality. This constraint is represented by a minimum fiber number, *N*_min_, and is related to fiber surface area (Equation 3). Hepatocytes depend on anchorage for maintenance of their function and phenotype (Lee et al., 2008; Morelli et al., 2007).

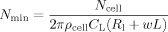
3

For a given set of operating conditions and geometric parameters for a BAL, the number of hollow fibers required to produce a viable design is defined by the range between *N*_min_ and *N*_max_. This concept was explored previously (Davidson et al., 2010), but here is extended to examine metabolic zonation for those viable designs with fiber numbers in the range of *N*_min_ to *N*_max_.

### Zonation Equations

As stated previously, oxygen is a primary modulator of liver zonation. As a result the position of the periportal (proximal to branches of the hepatic artery and portal vein), pericentral (central portion of the liver lobule) and perivenous (proximal the central vein) zones can be defined by the local oxygen tensions. Our starting assumption is that each of the three zones occupies an equal fraction of the cell mass (Rappaport, 1976). In vivo, blood enters the liver lobule containing oxygen at approximately 60–65 mm Hg which falls to 30–35 mm Hg in the perivenous zone (Jungermann and Kietzmann, 2000). This is a difficult to replicate with fractionated blood plasma or culture medium as they do not contain hemoglobin. As a result, oxygen levels tend to be higher at the inlet and lower at the outlet of the bioreactor than in the liver lobule. This wider oxygen range still tends to produce in vitro zonation similar to the native liver lobule (Allen and Bhatia, 2003; Allen et al., 2005). The periportal zone has been defined to exist where oxygen tensions are above 60 mm Hg, the pericentral zone occupies the range from 35 to 60 mm Hg and the perivenous zone experiences oxygen tensions below 35 mm Hg (Chen and Palmer, 2009; Sullivan et al., 2007; Sullivan and Palmer, 2006). The same definitions will be applied here.

**Figure 2:**
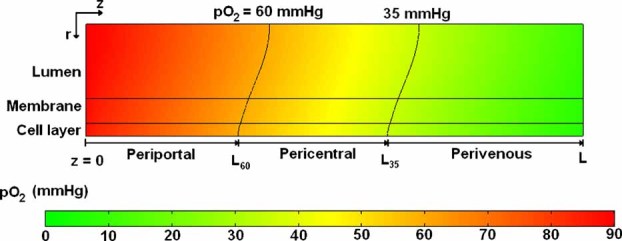
Definition of the three metabolic zones within a Krogh cylinder model of a BAL. The boundaries of each metabolic zone are indicated.

The fractional size of each zone is defined as the proportion of the axial distance between its boundaries to the total fiber length (Fig. 2). The axial distance to the zone boundary is proportional to the flow velocity (*L*_x_ = *k*_x_ū). A MATLAB function was written to solve the model using Comsol algorithms over a range of flow velocities and fiber lumen radii. This enabled the empirical relationships between zone boundary position and fiber lumen radius *R*_l_ to be derived. In turn this allows the fractional zone size to be expressed as a function of plasma flow rate *Q*, fiber number *N* and fiber geometrical parameters *R*_l_ and *L* as below, where the periportal zone size is defined (Equation 4).

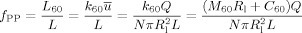
4

Here *f*_pp_ is the fractional size of the periportal zone, *L*_60_ is the axial distance along the fiber to the boundary of the periportal zone (the subscript 60 corresponding to 60 mm Hg pO_2_) and *L* is the length of the fiber. The proportionality coefficient *k*_60_ (units of s) can be represented as a linear function of fiber lumen radius *R*_l_ with a slope of *M*_60_ and an intercept of *C*_60_ (units of s/m and s respectively). The number of fibers in the bundle can vary between the maximum and minimum constraints (Equations 2 and 3). The procedure used here to define the periportal zone size in Equation 4 is also applied to define the pericentral and perivenous fractional zone sizes (see Supplementary Material).

From the definition of the zone sizes, it is possible to find the device flow rate *Q* that will allow the periportal and perivenous zones to occupy the same volume (though not necessarily the same volume as the pericentral zone). This parameter is named *Q*_eq_ as it represents a configuration where periportal and pericentral hepatic functions have equal prevalence. Whether this is actually an optimal situation for the BAL is a point discussed later. By rearranging the zone size equations so that the periportal and perivenous fractional zone sizes are equal, an expression for *Q*_eq_ is found (Equation 5).

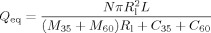
5

If this value of flow rate is below 300 mL/min—the technical limit of plasma flow rate in BAL applications (Pless and Sauer, 2005), then a BAL can designed where the periportal and pericentral metabolic zones occupy an equal amount of the cell volume.

### Parameter Values

Parameter values for the model were chosen so as to represent typical hollow fiber BAL systems represented in the literature (Table I). The ranges of fiber lumen radius and length were chosen so as to represent common hollow fiber geometries. The plasma flow rate (*Q*) range was determined by two factors: the maximum flow rate due to technical reasons (300 mL/min), and the minimum flow rate (200 mL/min) that can produce adequate operating region sizes from our previous work (Davidson et al., 2010). These flowrates are similar to those expected in the liver in vivo. A range of cell numbers was chosen around an intermediate value of 10 billion cells, this number is generally considered the minimum required for a functional BAL (Allen et al., 2001; Sullivan et al., 2007). The inlet partial pressure of oxygen was varied between 70 and 110 mm Hg. The lower end of the scale is similar to physiological arterial oxygen tension while the upper end approximately corresponds to the maximum solubility in plasma at 37°C. The standard value for maximal oxygen uptake rate *V*_max_ was equal to a value reported for hepatocytes in a similar system (Nyberg et al., 1994). This value falls within the range of oxygen consumption rates (OCR) reported for 3D hepatocyte cultures and summarized by Patzer (2004). The OCR units of mol/cell/s were converted by assuming that each cell had the same volume as a sphere of 25 µm diameter. At the lower end of the scale *V*_max_ was equal to 2.93 µM/s and at the upper limit was equal to 11.25 µM/s. This range was considered for the model. A value of 3 mm Hg was used for the Michaelis–Menten constant *K*_m_ (Hay et al., 2001). As hepatocytes form a layer one or two cells thick around each sinusoid (Telford and Bridgman, 1995), the effect of having a double cell layer around each hollow fiber was studied.

**Table I:** Parameter values considered in the model, with references for the ranges.

Parameter	Studied range	Standard value	Refs.
Fiber lumen radius *R*_l_	100–300 µm	200 µm	Hay et al. (2000, 2001), Sullivan et al. (2007)
Fiber length *L*	15–35 cm	25 cm	Hay et al. (2000), Moussy (2003)
Plasma flow rate *Q*	200–300 mL/min	250 mL/min	Pless and Sauer (2005)
Inlet partial pressure of oxygen pO_2_(in)	70–110 mm Hg	90 mm Hg	Sullivan et al. (2007)
Maximal oxygen uptake rate *V*_max_	2.93–11.25 µM/s	5.87 µM/s	Patzer (2004), Sullivan et al. (2007)
BAL cell population *N*_cell_	7.5–12.5 billion	10 billion	Allen et al. (2001)
Cell layer number *C*_L_	1–2	1	Saxena et al. (2003)

## Results

**Figure 3:**
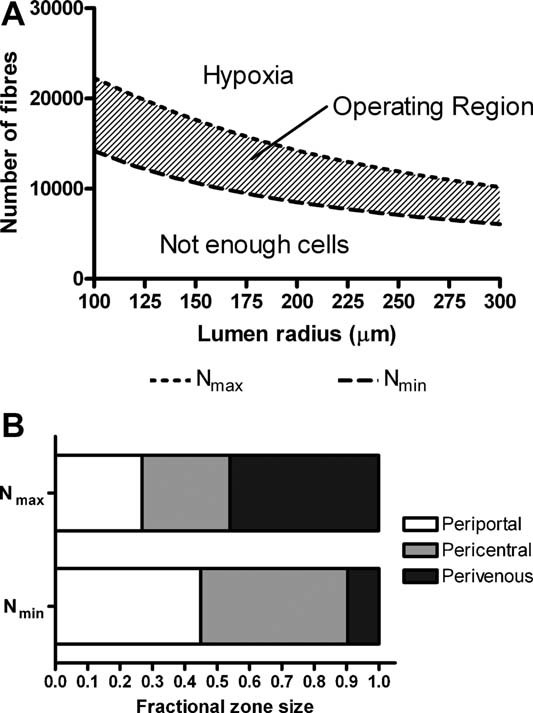
A : Operating region chart, produced in a previous study using the parameters of Table I. B: The distribution of the metabolic zones at both extremes of the operating region, for a fiber radius of 200 µm.

Figure 3A shows an operating region chart as described in our previous work (Davidson et al., 2010). It has been constructed using the standard parameter values in Table I for a range of fiber lumen radii *R*_l_. The top line, or maximum fiber number (*N*_max_) limit, defines how many fibers can be contained within the BAL while maintaining adequate hepatocyte oxygenation. The minimum fiber number (*N*_min_) limit defines the minimum number of fibers required to provide the BAL with an adequate cell population. Figure 3B displays how the metabolic zones are distributed in a BAL being operated under the same conditions for a fiber lumen radius of 200 µm. The chart shows the fractional zone sizes at the limits of fiber number (*N*_min_ and *N*_max_) and hence the extremes of zonal distribution while staying within the BAL operating region. The effects of varying the design parameters on the zone distributions are explored in the following section. In addition, values of plasma flow rate which minimize the difference in size between the periportal and perivenous zones (Equation 5) are plotted where possible.

### Influence of Fiber Dimensions

**Figure 4:**
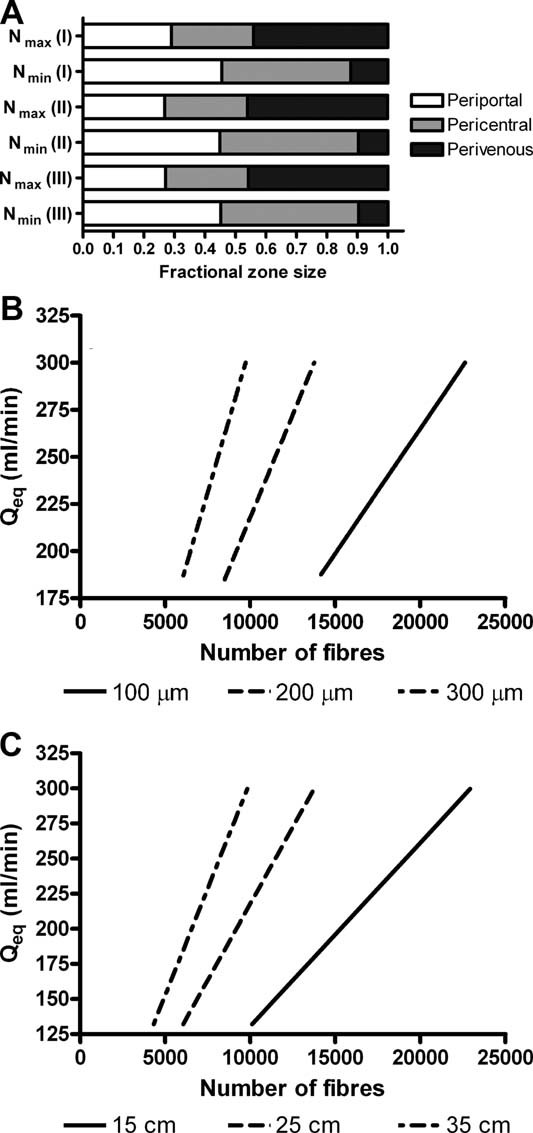
A : Distribution of metabolic zones in the case of (I) *R*_l_ = 100 µm, (II) *R*_l_ = 200 µm, and (III) *R*_l_ = 300 µm. B: The flow rate required to produced balanced periportal and perivenous zones (*Q*_eq_) is shown as a function of fiber number and (C) fiber length.

The significance of the hollow fiber lumen radius to the zone distribution in the BAL can be observed in Figure 4. The influence appears to be minimal with the distribution changing little over the range of fiber radii considered (Fig. 4A). When operating with the plasma flow rate *Q*_eq_ (Fig. 4B), the periportal and perivenous zones each occupy 34.1%, 33.2%, and 32.9% of the cell volume for lumen radii of 100, 200, and 300 µm respectively. Fiber length does not have any influence upon zone distribution and hence the zone distribution chart is not shown. While this may seem counterintuitive, changes to fiber length are offset by the corresponding changes in *N*_min_ and *N*_max_ such that the effect is canceled out. However, fiber length will have an influence on *Q*_eq_, the plasma flow rate that balances the metabolic zone distribution (Fig. 4C). In each case of fiber length, a flow rate equal to *Q*_eq_ will cause the periportal and perivenous zones to occupy 33.2%.

### Influence of Plasma Flow Rate

**Figure 5:**
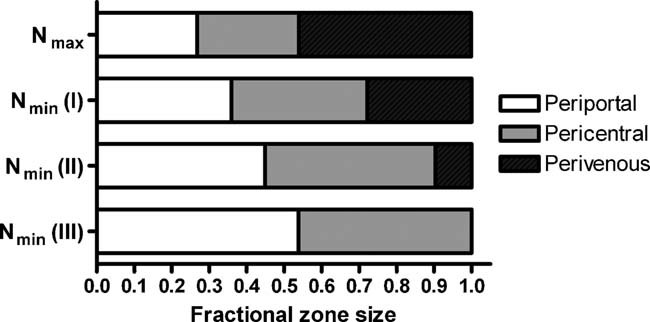
Distribution of metabolic zones in the case of (I) *Q* = 200 mL/min, (II) *Q* = 250 mL/min and (III) = 300 mL/min. The zone distribution is unaffected by *Q* when operating on the *N*_max_ limit and hence the bar is drawn only once.

Figure 5 shows how plasma flow rates in the BAL affect the relative size of each metabolic zone. The zone distribution is unaffected if the BAL is operated on the upper limit constraint, that is, the number of fibers *N* is equal to *N*_max_ and oxygen levels are just sufficient. In this case the periportal, pericentral and perivenous zones occupy 26.8%, 27.1%, and 46.0% of the cell volume respectively. At the other extreme where *N* = *N*_min_, increasing flow rate tends to increase the relative sizes of the periportal and pericentral zones. The periportal zone occupies a maximum of 53.8% of the BAL for the highest flow rate while the perivenous zone disappears entirely in this case.

### Influence of Inlet Oxygen Tension

**Figure 6:**
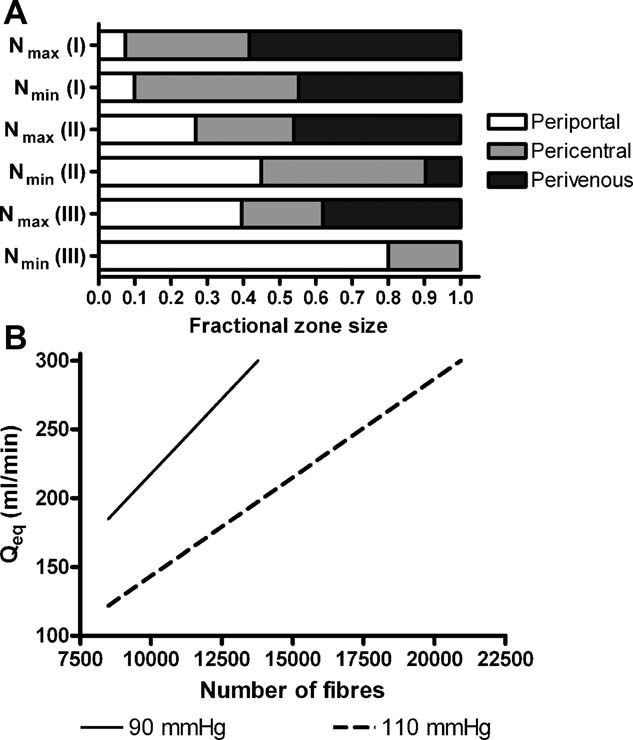
A : Distribution of metabolic zones in the case of (I) pO_2_(in) = 70 mm Hg, (II) pO_2_(in) = 90 mm Hg and (III) pO_2_(in) = 110 mm Hg. B: In the *Q*_eq_ plot, a pO_2_(in) value of 70 mm Hg required a *Q* value greater than 300 mL/min to produce equal periportal and pericentral zones and hence this was not included in the chart.

The distribution of each metabolic zone is under differing inlet oxygen tensions can be seen in Figure 6A. At a lower level of 70 mm Hg the perivenous zone is largest, occupying between 44.8% and 58.3% of the cell volume depending on the number of fibers used. When a higher oxygen tension of 110 mm Hg is employed, the perivenous zone shrinks in size and in the case of minimum fiber number it disappears entirely. The periportal zone dominates in this situation, occupying 80.0% of the cell volume. When using an inlet oxygen partial pressure of 70 mm Hg the flow rate required to produce equal periportal and perivenous zones is above 300 mL/min within the fiber number constraints (i.e., within the operating region), therefore it is impossible to achieve an equal zone distribution in the BAL for this partial pressure. For a given number of fibers in the BAL, a higher partial pressure of oxygen allows equal zones to be achieved (33.2% each for 90 mm Hg, 38.9% each for 110 mm Hg) with a lower device flow rate (Fig. 6B). A minimum flow rate of 122 mL/min can create an equal zone distribution in the case of pO_2_(in) = 110 mm Hg.

### Influence of Cell Number

**Figure 7:**
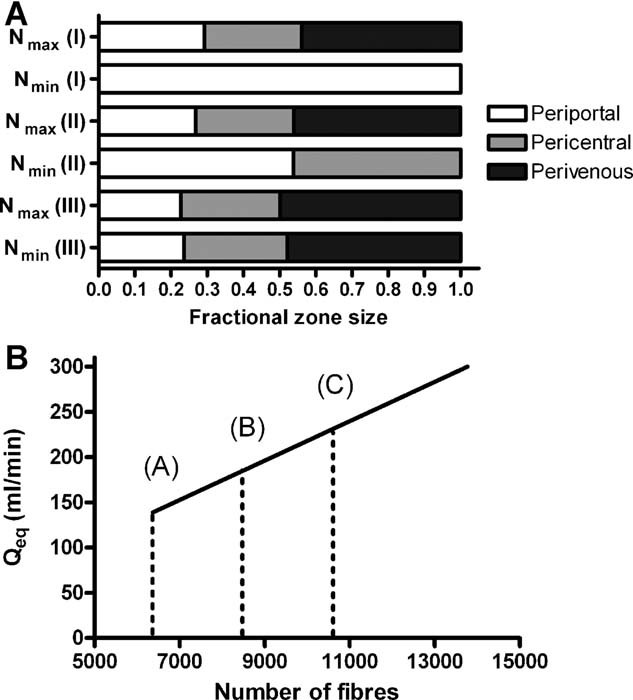
A: Distribution of metabolic zones in the case of (I) *N*_cell_ = 7.5 billion, (II) *N*_cell_ = 10.5 billion and (III) *N*_cell_ = 12.5 billion cells. B: *Q*_eq_ is plotted up to a limit of 300 mL/min for each of these *N*_cell_ values and the lines produced are co-incident. The vertical dash lines indicate the position of *N*_min_ for each of the three cases.

As was the case for flow rate, the cell number does not affect the distribution of zone sizes when the BAL is operated on the *N*_max_ constraint (Fig. 7A). However, when the BAL is operated with the minimum number of fibers required to satisfy the cell number constraint, the perivenous zone increases from 0% to 27.8% of the cell volume when the highest cell number is inoculated. To produce a zone distribution where the periportal and perivenous zones both occupy 33.2% of the cell volume each, the BAL should be operated with a plasma flow rate *Q*_eq_, given in the chart as a function of device fiber number (Fig. 7B). The contour is unaffected by the cell population size, though the lower limit of fiber number *N*_min_ will change.

### Influence of Maximum Oxygen Uptake Rate

**Figure 8:**
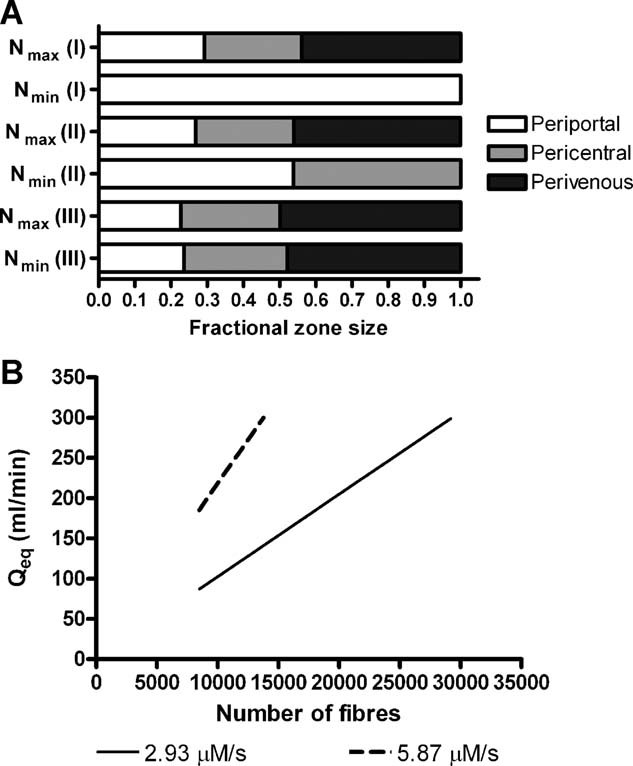
A : Distribution of metabolic zones in the case of (I) *V*_max_ = 2.93 µM/s, (II) *V*_max_ = 5.87 µM/s, and (III) *V*_max_ = 11.25 µM/s. B: For a *V*_max_ value of 11.25 µM/s, it was not possible to create an equal zone distribution for a *Q* value of less than 300 mL/min and hence that line is not included on the *Q*_eq_ chart.

Various values of maximum oxygen uptake rate *V*_max_ have been reported for hepatocytes so the significance of this parameter was examined as seen in Figure 8. A valid operating region could not be produced for a *V*_max_ value of 11.25 µM/s for the standard plasma flow rate value in Table I (250 mL/min), so a *Q* value of 300 mL/min was considered instead. When using the lowest *V*_max_ value of 2.93 µM/s, the entire BAL consists of periportal hepatocytes when operating on the *N*_min_ limit. When *V*_max_ is equal to 11.25 µM/s the perivenous zone is the largest in the BAL, occupying 47.9–49.9% of the cell volume depending on the fiber number (Fig. 8A). For this reason it was impossible to produce an equal zone distribution for the upper limit *V*_max_ value with a *Q*_eq_ value of less than 300 mL/min. A lower *V*_max_ value allows equal zone distribution to be achieved with a lower plasma flow rate. In the case of *V*_max_ equal to 2.93 µM/s, a minimum flow of 87 mL/min could produce periportal and pericentral zones each occupying 34.2% of the cell volume (Fig. 8B).

### Influence of Single or Double Cell Layer

**Figure 9:**
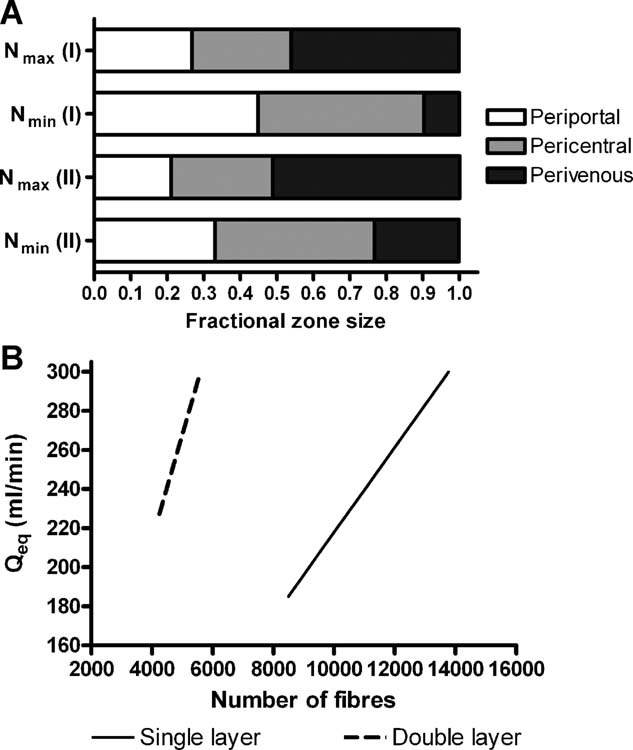
A : Distribution of metabolic zones in the case of (I) a single cell layer and (II) a double cell layer model. B: The *Q*_eq_ plot for each case is also shown.

Hepatocytes can be cultured as either a single or double layer on each hollow fiber, and the effect on the zone distribution can be seen in Figure 9A. The addition of an extra cell layer per fiber causes a gradual reduction in the size of the periportal and pericentral zones, accompanied by a corresponding increase in the perivenous zone size. The periportal zone fractional size at the *N*_max_ limit reduces from 26.8% for a cell monolayer to 22.1% for a double layer, or from 44.9% to 33.1% at the *N*_min_ limit. Conversely, the perivenous zone size increases from 46.0% to 51.2% of the cell volume at the *N*_max_ limit or from 9.7% to 23.1% at the *N*_min_ limit. For a monolayer system, operating at *Q*_eq_ (Fig. 9B) will produce periportal and perivenous zones of 33.2% fractional volume whereas they will each occupy 30.1% in the double layer system.

## Discussion

A mathematical model of oxygen transport within a bioartificial liver was set up and solved using finite element methods. The results of these simulations were used to define operating regions within which the BAL could adequately oxygenate entirely a set cell population. Within a viable BAL design as defined by the operating region, the oxygen tensions within the bioreactor delineate three metabolic zones. This article describes how altering the design parameters of the BAL affect the size of each of these zones within the operating region.

The effects of hollow fiber lumen radius and length were illustrated in Figures 4 and 5. It can be seen that lumen radius has very little effect on zonal distribution, while fiber length has no influence. These parameters are important, however, in forming the operating regions and hence determining the values of *N*_min_ and *N*_max_. In our previous work we showed that it was preferable to use numerous, shorter and thinner hollow fibers as opposed to fewer larger and longer fibers as this tended to produce wider operating regions and hence offer greater design flexibility and margin for error in the model (Davidson et al., 2010).

A key parameter in the design of a BAL is the flow rate of plasma from the patient, into the device. As would be expected, increasing the flow rate of oxygen-carrying plasma into the BAL will increase the proportion of periportal hepatocytes. However, a risk arises as a result of using high flow rates: the perivenous zone can become very small or disappear entirely. If the BAL is over-oxygenated, important functions related to the perivenous zone could become impaired. This situation highlights the need to mimic the in vivo oxygen gradient as closely as practically possible.

The inlet oxygen tension to the BAL was previously chosen to be 90 mm Hg as this is approximately the same as arterial oxygen levels and was able to adequately oxygenate the BAL in most cases. In vivo however the liver has a dual blood supply from the hepatic portal vein and hepatic artery. Around two-thirds of the blood supply is venous and hence hepatocytes actually experience oxygen tensions between 60 and 65 mm Hg, some way below 90 mm Hg (Jungermann and Kietzmann, 2000). The presence of hemoglobin in the bloodstream makes this possible without cellular necrosis occurring due to hypoxia. As hemoglobin is absent in the model, it is necessary to operate with higher-than-physiological oxygen tensions. The periportal zone is small in the case of 70 mm Hg inlet oxygen tension whereas it dominates the cell volume when the plasma is oxygenated to 110 mm Hg. An inlet oxygen tension of 70 mm Hg is too low to operate the BAL and hope to achieve equal zone distributions as flow rates above the limit of 300 mL/min would be required. On the other hand, maximizing the oxygen content of the plasma appears to offer more flexibility in controlling the size of the metabolic zones. A minimum flow rate of 122 mL/min is required to balance the zones in the case of inlet oxygen tension being 110 mm Hg if the BAL is operated with the parameters of Table I. The duty of the plasma pump in the BAL can be reduced through use of higher oxygen tensions, though whether the hepatocytes will function to the same degree at non-physiological oxygen tensions is another question. There is evidence that high oxygen tensions can damage hepatocytes (Miyazaki et al., 1991).

The number of cells required in the BAL is subject to debate, with the patient's residual liver function being a factor. Generally it is regarded that the minimum number of hepatocytes should be 10% of the cell mass in the liver, corresponding to approximately 10 billion cells (Allen et al., 2001; Sullivan et al., 2007). A range of cell numbers around this figure was examined to see how this parameter affected the zone distribution. Operating with a smaller number of cells corresponds to a lower required fiber number (*N*_min_) and hence the range of viable fiber numbers increases. This results in a wider operating region and hence a greater degree of selectivity in regards to zonal distribution. For the higher cell number of 12.5 billion, the range of viable fiber numbers is significantly reduced. As a result less control exists over the distribution of the metabolic zones though according to the chart for *Q*_eq_ a plasma flow rate from 230 to 300 mL/min will cause each of the metabolic zones to occupy approximately one-third of the cell volume, depending on the fiber number. This would indicate that a greater number of cells could reside in the BAL while still maintaining equal zone distribution. According to our calculations (not presented), up to 16.2 billion cells could be supported in the device while maintaining equal zone distribution and limiting plasma flow rate to 300 mL/min. The parameters of Table I are used to calculate this value.

In modeling oxygen uptake by hepatocytes, one parameter that is difficult to accurately identify is the maximum rate of oxygen uptake. It is very sensitive to culture conditions and a range of values have been reported in the literature, from 24 to 900 amol/cell/s (Patzer, 2004). For the higher value studied here it was found that viable operating regions could only be defined when the maximum flow rate of 300 mL/min was used. For a low *V*_max_ it is possible that the entire cell volume can be occupied by the periportal zone, an undesirable situation which would affect the useful functions of the BAL. However a lot of control does exist over the zone distribution due to the wide operating region, and a lower inlet flow rate or oxygen tension could compensate for the lower oxygen demand. On the other hand, if the hepatocytes have a high oxygen demand there is almost no control over the zone distribution. It would be necessary to increase the plasma oxygen content or reduce the number of cells inoculated in the BAL in order to more evenly distribute the zones. As stated above, the maximum flow rate is being used at this point. In a real system it is likely that the oxygen demand will vary transiently, beginning high and then decreasing as the cells adapt to their new environment (Patzer, 2004).

The mathematical model from our previous work (Davidson et al., 2010) on operating regions in a BAL system was modified to account for cells being arranged as a double layer on each fiber as opposed to solely being monolayers. By increasing the number of cells surrounding each fiber, fewer fibers are required in the BAL which is beneficial from a manufacturing point of view. However, it can be seen in the results above that by increasing the number of hepatocyte layers around each hollow fiber, the relative size of the perivenous zone increases. The periportal zone shrinks while the pericentral zone remains relatively unchanged in size. As a result, the minimum flow rate required to achieve equal zone distribution is greater in the double layer system, 227 mL/min opposed to 185 mL/min in the monolayer model.

It has been assumed that an equal distribution of the periportal, pericentral, and perivenous zones would be optimum in the device (Rappaport, 1976) and that the cells have been evenly distributed within the HFBR. For this reason, charts were produced that give values of plasma flow rate that will cause the periportal and perivenous zones to occupy the same proportion of the cell volume. However, perhaps the relative importance of each of the liver functions should be established before making this conclusion. For example, ammonia clearance takes place primarily in periportal hepatocytes whereas xenobiotic metabolism is typically seen in the perivenous zone. If ammonia clearance could be achieved through artificial means such as adsorption or filtration in series with the BAL (as seen in several hybrid devices (Gerlach et al., 1994; Rozga et al., 1992; Xue et al., 2001)), then it may be beneficial to increase the proportion of perivenous hepatocytes that detoxify drug molecules. Further experimental and anecdotal evidence is required to establish this hypothesis.

## Conclusions

Oxygen is a primary regulator of liver cell heterogeneity and hence the coordination of the various liver functions. In order to design a bioartificial liver that can function in a similar manner to the in vivo organ, physiological gradients of oxygen tension should be replicated as closely as possible within the extracorporeal device. Using a mathematical model and operating parameters that satisfy both oxygenation and cell number constraints, it was possible to see how each parameter was significant in controlling the distribution of the metabolic zones. As would be expected, increasing oxygenation of the BAL through higher flow rates and inlet oxygen tensions allows a greater range of zone sizes. However, it is possible to over-oxygenate the hepatocytes in a way that the perivenous zone is either small or absent entirely, potentially impairing the function of the BAL. Conversely, when using higher cell loadings or cells with higher oxygen demands the periportal zone can be reduced in size. This study shows how it is possible to control the distribution of the liver metabolic zones, while remaining within the operating limits of the BAL design. By making these considerations, the efficacy of BAL designs should improve and take a further step towards clinical use.

## Nomenclature

*c*: concentration of oxygen (mol/m^3^)
*C*_x_: empirical constant, relating *k*_x_ to *R*_l_ (s)
*C*_L_: number of cell layers surrounding each hollow fiber
*D*_hep_: diffusion coefficient for oxygen in hepatocyte layer (m^2^/s)
*D*_plasma_: diffusion coefficient for oxygen in plasma (m^2^/s)
*f*_PP/PC/PV_: size of each liver zone expressed as a fraction of the cell volume
*k*_l_: proportionality constant between *L*_x_ and ū (s)
*L*: fiber length (m)
*L*_x_: axial position of partial pressure contour (m)
*M*_x_: empirical constant, relating *k*_x_ to *R*_l_ (s/m)
*N*: number of fibers
*N*_cell_: number of cells in the BAL
*N*_max_: maximum allowable number of fibers according to the “no hypoxia” constraint
*N*_min_: minimum allowable number of fibers according to the cell number constraint
pO_2_: partial pressure of oxygen (mm Hg)
pO_2_(in): partial pressure of oxygen at the entrance of the BAL (mm Hg)
*Q*: plasma flow rate to the BAL (mL/min)
*r*: radial coordinate (m)
*R*_l_: fiber lumen radius (m)
*u*: fluid velocity vector (m/s)
ū: mean axial velocity (m/s)
*V*: generation/uptake term in the convection-diffusion equation (mol/m^3^/s)
*V*_max_: maximum oxygen consumption term in Michaelis–Menten equation (mol/m^3^/s)
*w*: thickness of hollow fiber membrane (m)
*x*: subscript denoting the relevant partial pressure of oxygen
*z*: axial coordinate (m)
