# Supplementary material for: A theoretical approach to zonation in a bioartificial liver
Source: Biotechnol Bioeng. 2012 Jan;109(1):234–43. doi: 10.1002/bit.23279 (PMC3579238; doi:10.1002/bit.23279)
Supplement: Supplementary file 1 [file bit0109-0234-sd1.doc]

*Davidson, Ellis, Chaudhuri; A Theoretical Approach to Zonation in a Bioartificial Liver*

**Supplementary Material**

Metabolic Zone Definitions

Pericentral zone

If the exit oxygen partial pressure is less than 35 mmHg, i.e. the perivenous zone does exist in the BAL:

If this is not the case then:

Perivenous zone

If the exit pO_2_ is less than 35 mmHg and hence the perivenous zone exists, its relative size is defined by:

Conversely if the exit oxygen partial pressure is above 35 mmHg, the perivenous zone size is zero:
